# High Prevalence of Antimicrobial Resistance and Integron Gene Cassettes in Multi-Drug-Resistant *Klebsiella pneumoniae* Isolates From Captive Giant Pandas (*Ailuropoda melanoleuca*)

**DOI:** 10.3389/fmicb.2021.801292

**Published:** 2022-02-03

**Authors:** Xia Yan, Xiaoyan Su, Zhihua Ren, Xueyang Fan, Yunli Li, Chanjuan Yue, Mei Yang, Huidan Deng, Youtian Deng, Zhiwen Xu, Dongsheng Zhang, Lin Li, Rong Hou, Songrui Liu, Junliang Deng

**Affiliations:** ^1^College of Veterinary Medicine, Sichuan Agricultural University, Chengdu, China; ^2^Sichuan Key Laboratory of Conservation Biology for Endangered Wildlife, Chengdu Research Base of Giant Panda Breeding, Sichuan Academy of Giant Panda, Chenghua, China

**Keywords:** giant panda, MDR *K. pneumonia*, ARGS, MGEs, integron

## Abstract

Multi-drug-resistant *Klebsiella pneumoniae* (MDR *K. pneumonia*) is increasingly being reported with corresponding increase in morbidity and mortality all over the world. However, limited information is available concerning MDR *K. pneumonia* in giant pandas. The objective of this study was to grasp the drug resistance profile of MDR *K. pneumonia* isolated from giant pandas. A total of 182 *K. pneumoniae* isolates were collected from fresh feces of 94 captive giant pandas of different ages and sex and separated by season. We performed a standard disk diffusion antimicrobial susceptibility test with the isolates and further evaluated the antibiotic resistance genes (ARGs) of multi-drug-resistant strains by high-throughput quantitative PCR. In addition, we then analyzed mobile genetic elements (MGEs), integron gene cassettes, and the multi-locus sequence typing of multi-drug-resistant strains by PCR. Antimicrobial susceptibility testing results demonstrated that a total of 30 (16.5%) *K. pneumoniae* isolates showed multiple drug resistances. The thirty MDR *K. pneumonia* isolates were mainly resistant to amoxicillin (100.0%), doxycycline (86.7%), chloramphenicol (60.0%), compound trimethoprim (60.0%) and trimethoprim (56.7%). Fifty different types of antibiotic resistance genes were found, which included a total of 671 antibiotic resistance genes, in the 30 multi-drug-resistant isolates. The top ten resistance genes were: vanTC-02, aacC, blaCTX-M-04, blaSHV-01, blaSHV-02, ampC-04, blaOXY, tetD, blaTEM and tetA-02. Thirteen mobile genetic elements were detected, of which IS26 (96.67%) and intI1 (96.67%) had the highest frequency. The thirty MDR K. pneumonia isolates were negative for the traA, traF, tnsA, IS1133, ISpa7, ISkpn6, intI2 and intI3 genes. Moreover, a further investigation of integrons revealed that two types of specific gene cassettes (dfrA12 + orfF + aadA2 and dfrA12 + orfF) were identified in class 1 integrons. Multi-locus sequence typing results showed that 22 STs in the thirty MDR *K. pneumonia* isolates were identified, the main type was ST37 (5/30). Our results illustrate that effective surveillance and strict biosecurity strategies should be taken to prevent the spread of multi-drug-resistant bacteria, and monitor the emergence of mobile genetic elements and integrons.

## Introduction

*Klebsiella Pneumonia* is a gram-negative *Enterobacteria*, and it is also an opportunistic pathogen responsible for an important proportion of nosocomial infections in human. Multi-drug-resistant *Klebsiella pneumoniae* (MDR *K. pneumonia*) was defined as being resistant to three or more antimicrobials (Dolejska et al., 2007; Magiorakos et al., 2012), and is increasingly being reported with corresponding increase in morbidity and mortality. MDR *K. pneumoniae* was more and more prevalent in the tertiary teaching hospital in western Kenya from 2002 to 2013 (Apondi et al., 2016), and MDR *K. pneumoniae* was an increasing cause of neonatal infections in India and other developing countries, with 60.86% of MDR *K. pneumoniae* infections leading to death (Jeena et al., 2001). Furthermore, researchers have detected MDR *K. pneumoniae* isolated from raw milk from dairy farms in Jiangsu and Shandong provinces in China recent years, which was a potential threat to food safety and public health (Yang et al., 2021).

Antimicrobial abuse is a serious risk for the emergence of MDR *K. pneumoniae*. In addition, horizontal gene transfer (HGT) *via* mobile genetic elements (MGEs) such as integrons, transposons, integrative conjugative elements, genomic islands, and plasmids are another important reason for the spread of antibiotic resistance genes (ARGs) carried by MDR *K. pneumonia* (Partridge et al., 2009; Chen et al., 2016). Integrons can capture, convert and adapt one or more resistance gene cassettes into functionally expressed genes *via* a self-efficient gene expression system. Meanwhile it can also simply transfer these genes between diverse bacterial species due to their linkage with plasmids (Gillings, 2014). Class 1, 2, and 3 integrons are the three main types of MGEs associated with antimicrobial resistance (Kaushik et al., 2018).

The giant panda (*Ailuropoda melanoleuca*) is one of the world’s most recognized and rarest animals and is now only distributed in the mountainous areas of Sichuan, Gansu, and Shanxi provinces in China (Wang et al., 2018). Giant pandas infected with *K. pneumoniae* have developed hemorrhagic enteritis, and died from hemorrhagic sepsis and subsequent cases of genital hematuria, and sepsis. Infection of *K. pneumoniae* has increased within the captive population of giant pandas (Wang et al., 2006; Sun et al., 2013; Lu et al., 2015), generally causes mixed infection with other bacteria, and has become one of the most important pathogens of captive pandas (Peng and Xiong, 2000). According to our investigation, for the past several decades, antibiotics were widely applied to prevent and treat bacterial infectious diseases in giant pandas (Guo L. et al., 2015; Yang et al., 2017), which may have led to the emergence of antibiotic resistant bacteria (ARB) and even multi-drug-resistant (MDR) bacteria. Our previous studies have confirmed the prevalence of drug-resistance in *K. pneumoniae* in captive giant pandas (Su et al., 2021). However, MDR *K. pneumoniae* isolates from giant pandas have gained little attention and characterization so far. To the best of our knowledge, this is the first report showing the baseline characteristics of antimicrobial resistance and integron gene cassettes in *K. pneumoniae* isolates from captive giant pandas. The study aims to characterize the antimicrobial resistance of 182 *K. pneumoniae* isolates collected from the feces of giant pandas of different ages and sex separated by season, and to study the ARGs and integron gene cassettes and other MGEs in 30 MDR *K. pneumonia*, and further analyze the impact of age, sex and season on the antibiotic resistance of 30 MDR *K. pneumonia.* This study will help improve the clinical treatment and *ex situ* conservation of giant pandas.

## Materials and Methods

### Samples

To investigate the prevalence of the antimicrobial resistance of *K. pneumoniae* isolates, 376 fresh fecal samples were collected from 94 captive giant pandas (female: *n* = 47, male: *n* = 47) housed at the Chengdu Research Base of Giant Panda Breeding (CRBGP) in Sichuan, China during 2020. Four fecal samples were collected from each individual giant panda during each season: spring (March to May), summer (June to August), autumn (September to November) and winter (December to February). All animals were considered healthy at the time and were managed under routine CRBGP husbandry practices. A total of 182 *K. pneumonia* isolates were identified from the fecal samples by Gram staining, 16s rDNA, and bacterial biochemical identification. Isolates were separated and analyzed by the sex of the panda (female samples: *n* = 107, male samples: *n* = 75), the season of sampling (spring: *n* = 62, summer: *n* = 80, autumn: *n* = 29 and winter: *n* = 11), and divided into three age groups [sub-adult (aged 1.5–5 years): *n* = 67, adult (aged 5–19 years): *n* = 92, and geriatric (aged 20 years or older): *n* = 23]. Confirmed *K. pneumonia* strains were stored in Luria-Bertani (LB) broth containing 20% glycerol at –80°C for further analyses.

### Antimicrobial Susceptibility Test of the *Klebsiella pneumonia* Isolates

The susceptibilities of all isolates to 24 antimicrobials were tested using the standard disk diffusion method recommended by the (Clinical and Laboratory Standards Institute [CLSI], 2020). The following seven classes of antibiotics of antimicrobial disks were used (Hangzhou Microbial Reagent Co., Ltd., China): β-lactams: piperacillin (PIC, 100 μg), amoxicillin (AML, 20 μg), cefotaxime (CTX, 30 μg), cefazolin (CEZ, 30 μg), ceftriaxone (CRO, 30 μg), cefuroxim (CXM, 30 μg), cefaclor (CEC, 30 μg), ampicillin/sulbactam 1:1 (SAM, 10/10 μg), aztreonam (AZM, 30 μg), meropenem (MEM, 10 μg), imipenem (IPM, 10 μg); Aminoglycosides: kanamycin (KAN, 30 μg), gentamicin (GM, 10 μg), streptomycin (STR, 10 μg); Quinolones: ofloxacin (OFX, 5 μg), norfloxacin (NOR, 10 μg), levofloxacin (LEV, 5 μg), ciprofloxacin (CIP, 5 μg); Chloramphenicol: chloramphenicol (CHL, 30 μg); Macrolides: azithromycin (AZM, 15 μg); Tetracyclines: doxycycline (DOX, 30 μg), minocycline (MH, 30 μg); Sulfonamides: compound trimethoprim (SXT, 23.75/1.25), trimethoprim (TMP, 5 μg). Results were interpreted in accordance with (Clinical and Laboratory Standards Institute [CLSI], 2020) criteria. *Escherichia coli* ATCC25922 was used as a control. All the antibiotics selected in this study were based on the information collected from CRBGP veterinarians and recent studies on the resistance of giant pandas (Zhang et al., 2009; Guo L. et al., 2015; Chen et al., 2018; Zou et al., 2018; Zhu et al., 2020) and antibiotics commonly resistant to *K. pneumonia* in clinics (Gao L. et al., 2020; Gao Q. et al., 2020). MDR was defined as being resistant to three or more classes of antibiotics (Magiorakos et al., 2012; Zhu et al., 2020).

### DNA Extraction and High-Throughput Quantitative PCR for Antibiotic Resistance Genes Carried by the Multi-Drug-Resistant *Klebsiella pneumonia* Isolates

Total genomic DNA was extracted from the MDR *K. pneumonia* isolates based on the results of antimicrobial susceptibility test using the TIANamp Bacteria DNA kit (Tiangen Biotech, Beijing, China) according to the manufacturer’s instructions. DNA samples were stored at –20°C.

To evaluate the abundance of ARGs in different MDR *K. pneumonia* isolates, high-throughput quantitative PCR (HT-qPCR) of ARGs was performed by Yearth Biotech (Changsha, China) using the SmartChip Real-time PCR (Warfergen Inc., United States) as described previously with a slight modification (Gao Q. et al., 2020). A total of 83 primer sets (including a 16S rRNA gene primer sets, not listed, see Supplementary Table 1) were used. HT-qPCR data was preprocessed as described previously, for each primer set, amplifications efficiency beyond the range (90∼110%) were discarded, and amplification was confirmed with more than two positive replicates. The relative copy number of ARGs was calculated and transformed to absolute copy numbers by normalizing to 16S rRNA gene copy numbers which were quantified separately from the Wafergen platform.

### Detection of Mobile Genetic Elements Carried by the Multi-Drug-Resistant *Klebsiella pneumonia* Isolates

Twenty-one pairs of primers were designed to detect MGEs in the 30 MDR *K. pneumoniae* isolates by PCR (Table 1; Zhu et al., 2020). PCR amplification was performed in a total volume of 25 μL: 2 × Dream Taq Green PCR master mix (Thermo Fisher Scientific, United States) 12.5 μL, forward and reverse primer each 1 μL, DNA template 2 μL, and ddH2O 8.5 μL. And ddH2O was set as a negative control. The PCR reaction conditions consisted of 5 min at 95°C, followed by 30 cycles of 95°C for 30 s, annealing temperature for 30 s, 72°C for 30 s, and a final extension at 72°C for 10 min. PCR products were subjected to electrophoresis in a 1% agarose gel stained with 4S Red Pus (Sangon Biotech, Shanghai, China), visualized under ultraviolet light and photographed using a gel documentation system (Bio-Rad, Hercules, United States).

**TABLE 1:** Primers of MGEs used in the study.

Primers	Sequence (5′–3′)	Product (bp)	References
TraA	F:AAGTGTTCAGGGTGCTTCTGCGC	272	Zhu et al., 2020
	R:GTCATGTACATGATGACCATTT		
TbrC	F:CGGYATWCCGSCSACRCTGCG	255	Zhu et al., 2020
	R:GCCACCTGYSBGCAGTCMCC		
TraF	F:CGGTGATGATTTGCGAACGA	400	Zhu et al., 2020
	R:AGCATTCCGGTCGGCCTGTA		
TnpU	F:CCAACTGATGGCGGTGCCTT	403	Zhu et al., 2020
	R:GGTATGGTGGCTTTCGC		
tnpA/Tn21	F:ATGCCACGTCGTTCCATCCTGTCC	300	Zhu et al., 2020
	R:CGGGTCTGCTCCCGCTGGCC		
TnsA	F:GCAGCAGCCTTACAAGACGAG	406	Zhu et al., 2020
	R:GCCACATAGCGCAACTCCTCC		
MerA	F:GACCAGCCGCAGTTCGTCTA	462	Zhu et al., 2020
	R:GCAGCASGAAAGCTGCTTCA		
tnp513	F:ATGTCGCTGGCAAGGAACGC	240	Zhu et al., 2020
	R:GGGTTCGCTGCGAGGGATTGT		
ISCR1	F:ATGTCGCTGGCAAGGAACGC	240	Zhu et al., 2020
	R:GGGTTCGCTGCGAGGATTGT		
ISCR3/14	F:GGACTTCATTGCACGGATCGAAGC	339	Zhu et al., 2020
	R:TGCACGATGCCCAGCACCGGGCCA		
IS1133	F:AGTACAAAAGCTGTGAGATTTCAG	622	Zhu et al., 2020
	R:GATATTCATGAGCGCAATATTGGC		
ISEcp1	F:CTTCATTGGCATTGATAAGTTAG	299	Zhu et al., 2020
	R:TGTAGCATCGGTTTCCCAGTTTC		
ISpa7	F:TCAGGCCTTCATCGCTGCCATCAGG	300	Zhu et al., 2020
	R:TAGGCGTACAGTGCTCTTTCAACGCA		
IS26	F:ATGAAGCCATTCAAAGGCCGGCAT	387	Zhu et al., 2020
	R:TATGCAGCTTTGCTGTTACGACGG		
ISkpn6	F:GAAGATGCCAAGGTCAATGCCAGG	240	Zhu et al., 2020
	R:TCACAGATACGCCATTCGCCTCAG		
ISkpn7	F:ATGTTGACCCAGGAGCAAACCGTG	300	Zhu et al., 2020
	R:GAGGAAGGCGCGCAATAACGAGAG		
ISaba1	F:AATGATTGGTGACAATGAAG	372	Zhu et al., 2020
	R:ATGCAGCGCTTCTTTGCAGG		
IS903	F:GCAATACGCACGCTTTCAGGC	240	Zhu et al., 2020
	R:ACTGCACGGTTACGGTCTGCA		
intI1	F:TCTCGGGTAACATCAAGG	243	Zou et al., 2018
	R:GTTCTTCTACGGCAAGGT		
intI2	F:CACGGATATGCGACAAAAAGGT	233	Zou et al., 2018
	R:GTAGCAAACGAGTGACGAAATG		
intI3	F:AGTGGGTGGCGAATGAGTG	600	Zou et al., 2018
	R:TGTTCTTGTATCGGCAGGTG		
In1	F:GGCATCCAAGCAGCAAG	Variable	Zou et al., 2018
	R:AAGCAGACTTGACCTGA		
In2	F:CGGGATCCCGGACGGCATGCACGATTTGTA	Variable	Zou et al., 2018
	P:GATGCCATCGCAAGTACGAG		

### Detection of the Integrons and Associated Cassette Arrays Carried by the Multi-Drug-Resistant *Klebsiella pneumonia* Isolates

All the MDR *K. pneumonia* isolates were also screened for the presence of int11, intI2 and intI3 genes. Moreover, strains that were positive for the intl1 or intl2 or intI3 genes were then evaluated for gene cassettes by PCR (Table 1; Zou et al., 2018). The PCR amplification, PCR reaction conditions and the analyses for PCR products were the same as described in section “Detection of Mobile Genetic Elements Carried by the Multi-Drug-Resistant *Klebsiella pneumonia* Isolates.”

### Analysis of Clonal Relationship in the Multi-Drug-Resistant *Klebsiella pneumonia* Isolates Based on the Multi-Locus Sequence Typing

MLST was performed to investigate the clonal relationship of the 30 MDR *K. pneumonia* isolates by amplifying the seven standard housekeeping loci *gapA*, *infB*, *mdh*, *pgi*, *phoE*, *rpoB*, and *tonB* as described previously (Guo C. et al., 2015). The eBURST software was used to construct an evolutionary relationship map by analyzing the data of allelic sequence obtained in this study. That six of the seven sites were same was defined as a clone group. A type group containing 4 or more ST types was defined as a clonal complex (CC), of which one ST type was calculated to be the origin ST type, The other ST types was evolved based on the origin ST type. Compared with the original ST type, single-locus variant (SLV) has one different housekeeping gene locus. The single type does not belong to any clonal complex.

### Sequence and Data Analysis

All positive PCR products (MGEs, integrons and MLST) were sequenced by Sangong Biotech (Shanghai, China) in both directions. Sequences of MGEs and gene cassettes were analyzed online using BLAST,^1^ and sequences of MLST were analyzed online using MLST.^2^

### Statistical Analysis

Data were analyzed by a χ^2^-test using SPSS Statistics version 18.0. This test was used to compare the isolation rate of MDR *K. pneumonia* by sex in different age groups of the giant pandas by season. The difference of the number of antimicrobial resistances in MDR *K. pneumonia* strains from the individual giant pandas by sex, age and season groupings were tested with the “wilcox_test.” function in R package “rstatix.” The correlation between the antimicrobial resistant levels of MDR *K. pneumonia* and traits of the giant pandas by sex, age and season groupings using the Fisher’s test with “fisher. test” in “stats” R package. Differences among groups were considered significant at *P* < 0.05.

## Results

### The Isolation of the Multi-Drug-Resistant *Klebsiella pneumonia* Strains

**FIGURE 1:**
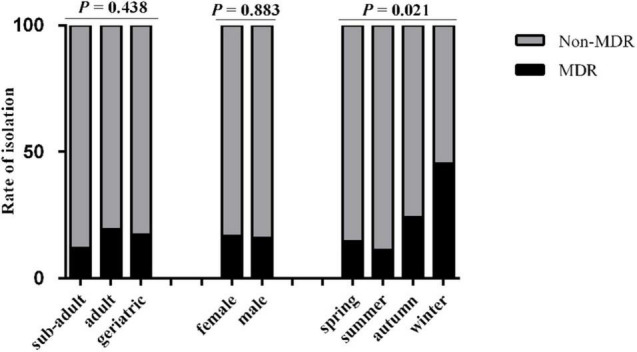
The rate of isolation of non-MDR and MDR *K. pneumonia* from giant pandas of different ages and gender in four seasons. Sub-adult: giant pandas aged 1.5–5 years; Adult: giant pandas aged 5–19 years; Geriatric: giant pandas aged 20 years or older. Spring, March to May; Summer, June to August; Autumn, September to November; Winter, December to February. Gray, the non-MDR *K. pneumonia* from giant pandas; Black, the MDR *K. pneumonia* from giant pandas. Data were analyzed by a χ^2^-test using SPSS Statistics version 18.0. *P* < 0.05: The age, sex and the season of sampling had a significant influence on the rate of isolation of non-MDR and MDR *K. pneumonia* from giant pandas.

A total of 182 *K. pneumonia* strains were isolated from the feces of captive giant pandas showing different degrees of resistance to 24 antimicrobials. Thirty strains which were identified as K1∼K30 were found to be multidrug resistant (MDR) (not listed, see Supplementary Table 2). The isolation rate of MDR *K. pneumonia* was 16.48% (30/182), of which the sub-adult group was 11.94% (8/67), adult group was 19.57% (18/92), and geriatric group was 17.39% (4/23), with the age of the giant panda having no significant effect on the isolation rate (*P* > 0.05). The isolation rate of MDR *K. pneumonia* was 16.82% (18/107) in female, and 16.00% (12/75) in male. There was no significant difference in the isolation rate based on the sex of the subject (*P* > 0.05); however, season had a significant effect on the isolation rate of MDR *K. pneumonia* (*P* < 0.05). The isolation rate was 14.52% (9/62) in spring, 11.25% (9/80) in summer, 24.14% (7/29) in autumn, 45.45% (5/11) in winter (Figure 1).

### The Comparison of Antimicrobial Resistances in Multi-Drug-Resistant Individual Giant Pandas

**FIGURE 2:**
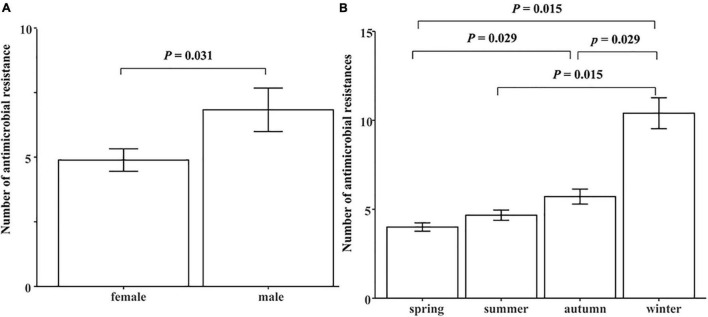
The comparison of number of antimicrobial resistances in 30 MDR giant pandas in different sex and season groups. **(A)** The effect of sex on the number of antimicrobial resistances in each MDR giant panda. **(B)** The effect of season on the number of antimicrobial resistances in each MDR giant panda. Spring, March to May; Summer, June to August; Autumn, September to November; Winter, December to February. Data were analyzed with the “wilcox_test.” function in R package “rstatix.” *P* < 0.05: The sex or the season had a significant effect on the number of antimicrobial resistances in each MDR giant panda.

We further analyzed the effects of age, sex and season on the number of antimicrobial resistances in each MDR giant panda. It was found that the antimicrobial resistances in MDR male giant pandas was significantly higher than that of females (*P* < 0.05) (Figure 2A). Additionally, there was a significantly increasing trend of the number of antimicrobial resistances from spring to winter. Compared with other seasons, the antimicrobial resistance of giant pandas in winter was significantly higher (*P* < 0.05) (Figure 2B). However, age was not significantly correlated to the number of antimicrobial resistances (*P* > 0.05).

### The Analysis of Antimicrobial Susceptibility in the Multi-Drug-Resistant *Klebsiella pneumonia* Isolates

**FIGURE 3:**
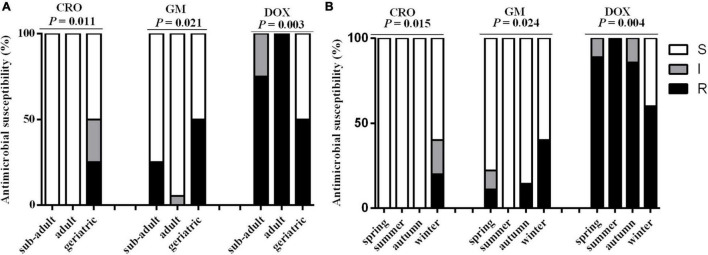
The comparison of antimicrobial susceptibility in the 30 MDR *K. pneumonia* isolates in different age and season groups. **(A)** The effect of age on antimicrobial susceptibility in MDR *K. pneumonia* isolates. Sub-adult: 1.5–5 years; Adult: 5–19 years; Geriatric: 20 years or older. **(B)** The effect of season on antimicrobial susceptibility in MDR *K. pneumonia* isolates. Spring, March to May; Summer, June to August; Autumn, September to November; Winter, December to February. CRO, ceftriaxone, GM, gentamicin, DOX, doxycycline. White, MDR *K. Pneumonia* isolates which were sensitive to antibiotics; Gray, MDR *K. Pneumonia* isolates which were intermediate to antibiotics; Dark, MDR *K. Pneumonia* isolates which were resistant to antibiotics. Data were analyzed with the “fisher. test” in “stats” R package. *P* < 0.05: The age or the season had a significant infect on antimicrobial susceptibility in MDR *K. pneumonia* isolates.

Thirty MDR *K. pneumonia* strains showed resistance to AML (100.00%, 30/30), DOX (86.67%, 26/30), CHL (60.00%, 18/30), SXT (60.00%, 18/30), and TMP (56.67%, 17/30); resistance to the remaining 19 antimicrobials was observed in less than 50% of the samples (Table 2). Fisher’s test showed that the antimicrobial resistance of MDR *K. pneumonia* strains to CRO, GM, and DOX was significantly related to age and season (*P* < 0.05) (Figure 3), while the remaining antimicrobials were not related to either factor (*P* > 0.05). Additionally, antimicrobial resistance of MDR *K. pneumonia* strains was not significantly correlated to sex of giant pandas (*P* > 0.05).

**TABLE 2:** Antimicrobial susceptibility of 30 MDR *K. pneumoniae* isolates from the feces of captive giant pandas in four seasons in China.

Name of giant pandas	Time of sampling	Strains	Age group	Sex group	Resistance profile
P1	Winter	K1	Geriatric	M	PIC/AML/CTX/CEZ/CRO/CXM/CEC/GM/STR/AZM/SXT/TMP
P6	Summer	K2	Adult	M	AML/CHL/AZM/DOX/SXT/TMP
P14	Summer	K3	Adult	F	AML/CTX/CHL/DOX
P4	Spring	K4	Adult	F	AML/CHL/DOX/MH
P4	Summer	K5	Adult	F	PIC/AML/CHL/DOX
P8	Spring	K6	Sub-adult	M	AML/CHL/DOX/MH
P4	Winter	K7	Adult	F	AML/STR/OFX/NOR/LEV/CIP/CHL/AZM/DOX/SXT/TMP
P6	Spring	K8	Adult	F	AML/CHL/DOX/SXT/TMP
P8	Autum	K9	Sub-adult	M	AML/IPM/CHL/DOX/MH/SXT/TMP
P12	Spring	K10	Adult	F	AML/MEM/STR//DOX
P2	Winter	K11	Geriatric	M	PIC/AML/CTX/CEZ/CXM/CEC/GM/STR/AZM/SXT/TMP
P9	Summer	K12	Adult	F	AML/IPM/CHL/DOX
P8	Summer	K13	Sub-adult	M	AML/CHL/DOX/SXT/TMP
P14	Spring	K14	Adult	F	AML/STR/DOX
P7	Spring	K15	Geriatric	F	AML/KAN/DOX/MH
P5	Winter	K16	Adult	F	AML/STR/CHL/DOX/MH/SXT/TMP
P15	Spring	K17	Sub-adult	F	AML/GM/SXT/TMP
P16	Autum	K18	Sub-adult	M	AML/GM/SXT/TMP
P13	Summer	K19	Adult	F	AML/AZM/LEV/DOX
P9	Autum	K20	Adult	F	AML/STR/DOX/MH/SXT/TMP
P3	Winter	K21	Adult	M	PIC/AML/CEZ/CXM/CEC/SAM/CHL/DOX/MH/SXT/TMP
P12	Summer	K22	Adult	F	AML/STR/DOX/SXT/TMP
P13	Spring	K23	Adult	F	AML/CHL/DOX
P3	Spring	K24	Adult	M	AML/CHL/DOX/SXT/TMP
P7	Autum	K25	Geriatric	F	AML/CHL/DOX/SXT/TMP
P16	Summer	K26	Sub-adult	M	AML/CHL/DOX/SXT
P15	Summer	K27	Sub-adult	F	PIC/AML/DOX/MH/SXT/TMP
P12	Autum	K28	Adult	F	AML/CHL/DOX/SXT/TMP
P10	Autum	K29	Sub-adult	M	AML/SAM/DOX/MH/SXT/TMP
P6	Autum	K30	Adult	M	AML/KAN/LEV/CHL/DOX/SXT/TMP

### The Antibiotic Resistance Genes Prevalence of the Multi-Drug-Resistant *Klebsiella pneumonia* Isolates

**FIGURE 4:**
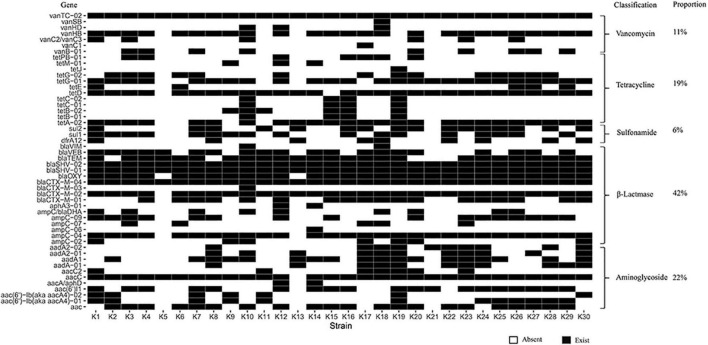
Heat-map for the ARGs in the 30 MDR *K. pneumonia* isolated from the feces of giant pandas. Columns: K1∼K30 mean 30 MDR *K. pneumoniae* isolates. Rows: 50 different types of ARGs, the classification and proportion. White: The *K. pneumoniae* isolate which did not carry the ARG; Black: The *K. pneumoniae* isolate which carried the ARG.

We extracted DNA from the 30 MDR *K. pneumoniae* isolates and analyzed ARGs. Our results showed an abundance of various ARG types in the 30 MDR *K. pneumoniae* isolates, where a total of 50 different types of ARGs were detected, including 671 ARGs. The top ten resistance genes were: vanTC-02, aacC, blaCTX-M-04, blaSHV-01, blaSHV-02, ampC-04, blaOXY, tetD, blaTEM and tetA-02. 671 unique ARGs had potential to confer resistance against a range of antibiotics such as β-Lactamase resistance (41.58%), aminoglycosides resistance (22.21%), vancomycin resistance (11.18%), tetracycline resistance genes (18.93%), sulfonamide resistance (6.11%) (Figure 4).

### The Mobile Genetic Elements Prevalence of the Multi-Drug-Resistant *Klebsiella pneumonia* Isolates

We extracted DNA from the 30 MDR *K. pneumoniae* isolates and analyzed MGEs. The results showed that 13 MGEs were detected among the 30 MDR isolates: IS26 (96.67%, *n* = 29), intI1 (96.67%, *n* = 29), tnpU (93.33%, *n* = 28), tnpA/Tn21 (93.33%, *n* = 28), IS903 (83.33%, *n* = 25), tbrC (36.67%, *n* = 11), ISEcp1 (30.00%, *n* = 9), merA (20.00%, *n* = 6), tnp513 (10.00%, *n* = 3), ISkpn7 (10.00%, *n* = 3), ISaba1 (10.00%, *n* = 3), ISCR3/14 (6.67%, *n* = 2), and ISCR1 (3.33%, *n* = 1). All isolates were negative for the traA, traF, tnsA, IS1133, ISpa7, ISkpn6, intI2, and intI3 genes (Table 3).

**TABLE 3:** MGEs and integron gene cassettes in 30 MDR *K. pneumoniae* isolates from the feces of captive giant pandas in China.

Strains	Components of mobile genetic elements	Integron	Gene cassettes
K1	tnpU + tnpA/Tn21 + ISEcp1 + IS26 + ISkpn7 + IS903 + intI1	Class1	
K2	tnpU + tnpA/Tn21 + ISEcp1 + IS26 + IS903 + intI1	Class1	
K3	tnpU + tnpA/Tn21 + merA + IS26 + IS903 + intI1	Class1	
K4	tnpU + tnpA/Tn21 + merA + IS26 + intI1	Class1	
K5	tnpU + tnpA/Tn21 + IS903 + intI1	Class1	
K6	tnpU + tnpA/Tn21 + IS26 + IS903 + intI1	Class1	
K7	tnpU + tnpA/Tn21 + tnp513 + IS26 + IS903 + intI1	Class1	
K8	tbrC + tnpU + ISCR3/14 + IS26 + IS903 + intI1	Class1	dfrA12 + orfF + aadA2
K9	tnpU + tnpA/Tn21 + merA + IS26 + IS903 + intI1	Class1	
K10	tnp513 + IS1133 + ISEcp1 + IS26 + IS903		
K11	tnpU + tnpA/Tn21 + ISCR3/14 + IS1133 + ISEcp1 + IS26 + IS903 + intI1	Class1	
K12	tbrC + tnpU + tnpA/Tn21 + IS1133 + IS26 + IS903 + intI1	Class1	
K13	tnpU + tnpA/Tn21 + IS26 + IS903 + intI1	Class1	dfrA12 + orfF
K14	tnpU + tnpA/Tn21 + merA + IS26 + intI1	Class1	
K15	tbrC + tnpU + tnpA/Tn21 + IS26 + IS903 + intI1	Class1	
K16	tnpU + tnpA/Tn21 + IS26 + intI1	Class1	
K17	tbrC + tnpU + tnpA/Tn21 + merA + IS26 + ISaba1 + intI1	Class1	
K18	tbrC + tnpA/Tn21 + tnp513 + ISCR1 + ISEcp1 + IS26 + ISkpn7 + intI1	Class1	dfrA12 + orfF
K19	tbrC + tnpU + tnpA/Tn21 + IS1133 + ISEcp1 + IS26 + ISkpn7 + ISaba1 + IS903 + intI1	Class1	
K20	tnpU + tnpA/Tn21 + IS1133 + IS26 + IS903 + intI1	Class1	
K21	tbrC + tnpU + tnpA/Tn21 + IS1133 + IS26 + IS903 + intI1	Class1	
K22	tbrC + tnpU + tnpA/Tn21 + IS1133 + IS26 + IS903 + intI1	Class1	dfrA12 + orfF + aadA2
K23	tnpU + tnpA/Tn21 + IS26 + IS903 + intI1	Class1	
K24	tnpU + tnpA/Tn21 + ISEcp1 + IS26 + IS903 + intI1	Class1	dfrA12 + orfF
K25	tbrC + tnpU + tnpA/Tn21 + ISEcp1 + IS26 + ISaba1 + IS903 + intI1	Class1	
K26	tbrC + tnpU + tnpA/Tn21 + ISEcp1 + IS26 + IS903 + intI1	Class1	
K27	tnpU + tnpA/Tn21 + IS26 + IS903 + intI1	Class1	
K28	tbrC + tnpU + tnpA/Tn21 + IS26 + IS903 + intI1	Class1	
K29	tnpU + tnpA/Tn21 + IS26 + IS903 + intI1	Class1	
K30	tnpU + tnpA/Tn21 + merA + IS26 + IS903 + intI1	class1	

### The Characterization of Integron Types and Gene Cassettes Carried by the Multi-Drug-Resistant *Klebsiella pneumonia* Isolates

We further studied integrons in the 30 MDR *K. pneumoniae* isolates: 96.67% (29 isolates) were positive for the intI1 genes, while intI2 and intI3 were not detected. We further analyzed antimicrobial resistance related to gene cassettes in the 29 intI1-positive isolates. The results showed that only in five strains were the drug resistance cassettes detected (dfrA12 + orfF + aadA2 in 2 isolates, and dfrA12 + orfF in 3 isolates) from the 29 intI1-positive isolates (Table 3).

### The Multi-Locus Sequence Typing Characteristics of the Multi-Drug-Resistant *Klebsiella pneumonia* Isolates

**FIGURE 5:**
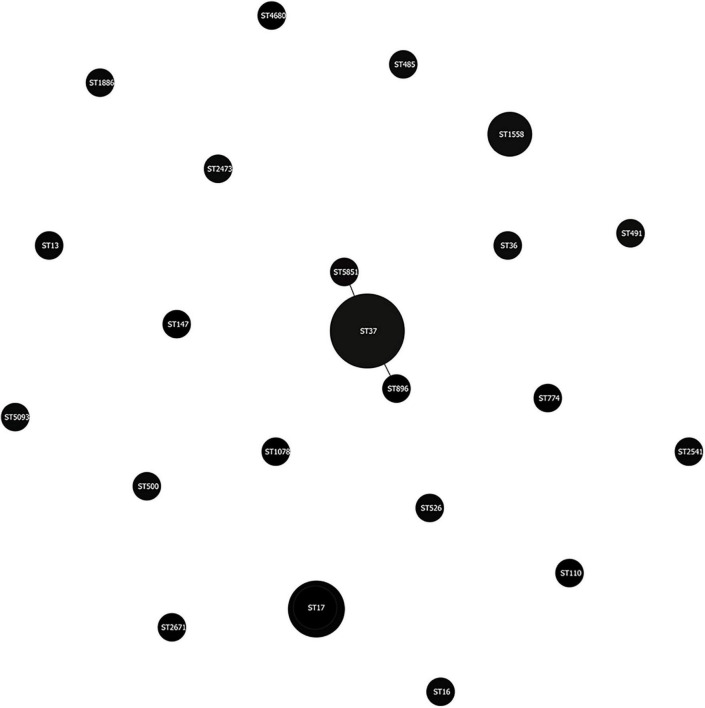
MLST-based clonal Structure of the 30 MDR *K. pneumonia* isolates from the feces of giant pandas. The scheme was constructed using eBURST analysis. STs were symbolized by dots, the size of a dot corresponded to the number of isolates belonging to an ST. SLVs (single-locus variant: has one different housekeeping gene locus compared with the original ST type) were linked by solid lines.

We use MLST typing to understand the clonal relationship of the 30 MDR *K. pneumonia* strains isolated between different giant pandas. Twenty-two STs were identified, of which five strains belonged to ST37, three strains belonged to ST17, and three strains belonged to ST1558. Clonal Structure analysis through eBURST software showed that thirty MDR *K. pneumoniae* isolates were highly diverse, but ST37 had two SLVs (Figure 5).

## Discussion

MDR *K. pneumoniae* was first reported in the United States, followed by Europe, South America, and Asia (Winokur et al., 2001). The present study is the first to detect MDR *K. pneumonia* isolated from captive giant pandas, with an isolation rate of 16.48%. At the same time, our data illustrated that the isolation rate of MDR *K. pneumonia* was not significantly related to the age or sex of the giant panda. The isolation rate of MDR K. *pneumonia* was found to be 30–70% in a hospital located in Central China (Guo et al., 2017), which was higher than our study in giant pandas. The reason for the lower isolation rate of MDR *K. pneumonia* in giant pandas may be explained by the possibility that antibiotics were used less frequently to treat giant pandas, in addition, *K. pneumoniae* strains in the study were isolated from feces of healthy giant pandas, while *K. pneumoniae* strains in other researches were isolated from hospital materials.

In this study, MDR *K. pneumonia* strains showed resistance to AMX (100.00%), DOX (86.67%), CHL (60.00%), SXT (60.00%), and TMP (56.67%). Zhu et al. (2020) also found *E. coli* strains isolated from feces of giant pandas showed resistance to AMX, DOX, CHL, and SXT, but the resistance rates of these antibiotics were all lower than 50%. Our resistance rates to AMX, DOX, CHL, and SXT were higher than those of *E. coli* isolated from captive giant pandas. The antibiotics in our study were mainly distributed in β-lactams and tetracyclines, which was consistent with previous studies on *E. coli* (Zhu et al., 2020). We further found some strains were resistant to more than 10 antibiotics in this study, even though CHL was banned for animals, resistance to CHL was observed in 60% of the MDR strains. For these reasons, it is important to be prudent in the use of antimicrobials with giant pandas, and the supervision of antimicrobial resistance in isolates from giant pandas can be useful in choosing antibiotics for preventing *K. pneumonia* infections. In addition, our results also found that among the 24 antibiotics, the drug resistance rates of CRO, GM, and DOX were significantly related to the age of the giant panda. The possible reason was that the disease susceptibility of giant pandas differ by age, and the selections of antibiotics for clinical treatment were also different. At the same time, we found that the antimicrobial susceptibility of MDR *K. pneumonia* isolated from the geriatric groups of giant pandas to CRO and GM was significantly lower than that of the adult groups and sub-adult groups, which indicated that more attention should be paid to the rational use of antibiotics for sick elderly pandas.

HT-qPCR enables a rapid and sensitive quantification of a number of ARGs, conferring resistance to almost all major classes of antibiotics and providing comprehensive profiles of ARGs (Su et al., 2015). In this study, fifty different types of ARGs were found, which included a total of 671 ARGs, which mainly belonged to β-Lactamase (41.58%), which was consistent with the findings of Hu et al. (2021). At the same time, the results of antimicrobial susceptibility test showed that the MDR isolates also had high resistance to β-lactam antibiotics, especially the resistance rate to AML, which was 100%. The resistance mechanism of *K. pneumonia* to β-lactam antibiotics is to mainly produce extended-spectrum β-lactamases encoded by extended-spectrum β-lactamase genes that destroy the β-lactam ring and lead to antibiotic inactivation (Fluit et al., 2001). Although β-lactam antibiotics and genes were diverse, the resistance phenotype and genotype of isolates roughly corresponded through this study. Given that rapid distribution and occurrence of novel ARGs among bacteria are intensifying problems throughout the world, it is necessary to monitor the resistance genes carried by the bacteria of giant pandas.

MGEs play an important role in the dissemination of ARGs among *K. pneumonia* strains. We had detected 13 MGEs among the 30 MDR isolates. The number of MGEs detected in this study was higher than the previous research on *E. coli*. strains isolated from giant panda, while traA, traF, tnsA, ISpa7, ISkpn6, and intI3 genes were all negative, which was consistent with previous findings (Zhu et al., 2020). The occurrence of MDR among microbes is considered to be associated with MGEs due to their ability to transfer several ARGs instantaneously (Martinez, 2009). Horizontal transfer of ARGs mediated by MGEs is one of the main mechanisms for the dissemination of ARGs (Chen et al., 2018). MGE as a carrier of ARGs plays an important role in capturing, accumulating and spreading, this can occur between cells of the same strain or between different *K. pneumonia* strains, enabling the rapid and widespread growth of antibiotic-resistant bacteria. In addition, the horizontal transfer of ARGs accelerates the emergence of drug-resistant and multi-drug-resistant bacteria in clinical practice (Chamosa et al., 2017). In this study, MDR *K. pneumonia* strains isolated from different giant panda carried some same ARGs, and a large number of MGEs were detected. Therefore, it can be speculated that there was the possibility of sharing the ARGs between giant pandas in this study. However, further investigation is needed to determine how the ARGs are shared.

We further analyzed antimicrobial resistance related to integrons in the 30 MDR *K. pneumonia* isolates. Integrons are genetic units consisting of determinants of a site-specific recombination event, which can mobilize and capture genes encoded on mobile elements known as gene cassettes (Gillings, 2014). In our study, the proportion of isolates containing the intI1 gene (96.67%, 29/30) was alarmingly higher than that previously reported in giant pandas (Zou et al., 2018; Zhu et al., 2020). We speculated the reason was that the intI1 gene was detected from MDR *K. pneumonia*. The diversity of gene cassettes are incorporated in the integrons and are able to encode antibiotics resistance to aminoglycoside, sulfonamides and trimethoprim. Accordingly, integrons were significantly connected with MDR (San et al., 2008; Tajbakhsh et al., 2015; Akrami et al., 2017). The intI2 and intI3 genes were not detected in our study. Many studies revealed that the class 1 integron was more prevalent than the class 2 or 3 in gram-negative bacteria (Lavakhamseh et al., 2016; Chamosa et al., 2017; Rehman et al., 2017; Sidhu et al., 2017). Moreover, class 3 integrons are reported to be rare in animals and clinical samples. Therefore, the presence of only intl1 integrons and the absence of intl2 and intl3 integrons in the present study were consistent with other research (Chen et al., 2010, 2016; Rehman et al., 2017; Zou et al., 2018; Zhu et al., 2020). Sequencing analysis showed that there were only two types of gene cassettes (dfrA12 + orfF + aadA2 in 2 isolates, and dfrA12 + orfF in 3 isolates) from the 29 intI1-positive isolates in this study. The other 24 strains provided negative results for the gene cassettes likely due to retained empty or undetermined intl1 VRs, which was also reported in other studies (Zhang et al., 2019; Zhu et al., 2020). Previous studies had confirmed that the gene cassettes dfrA12-orfF-aadA2 and dfrA17-aadA5 were dominant in giant pandas. These gene cassettes have previously been reported in *E. coli* isolates (Koczura et al., 2015; Yu et al., 2016; Zou et al., 2018; Zhu et al., 2020), which were constituted by trimethoprim resistance genes (dfrA12, dfrA17), and also carried the aadA2 and aadA5 genes which led to resistance to aminoglycoside (example: streptomycin). In addition, these gene cassettes can code a reading frame (orfF) for a hypothetical protein with an unknown function (Zhang et al., 2019). As far as we know, the integron gene cassettes in our study exist widely in humans, animals, plants, and the environment, however, this is the first report of integron gene cassettes in *K. pneumonia* isolated from the feces of giant pandas. It is emphasized that we should focus more on intergrons which contribute to the effective spread of ARGs.

MLST is an unambiguous procedure for characterizing isolates of bacterial species using the sequences of internal fragments of (usually) seven house-keeping genes (Jolley et al., 2018). In this study, thirty MDR *K. pneumoniae* isolates were divided into 22 STs, five of which belonged to ST37. Clonal Structure analysis through eBURST software showed that there was no clonal complex in thirty MDR *K. pneumoniae* isolates, but ST37 had two SLVs, which indicated that although MDR *Klebsiella pneumoniae* strains isolated from different giant pandas were highly diverse, some strains still had the same origin.

## Conclusion

In our study, 30 MDR strains among 182 *K. pneumonia* strains isolated from the feces of captive giant pandas. All of the MDR *K. pneumonia* strains carried a large number of ARGs and MGEs, especially 29 of 30 MDR *K. pneumonia* strains carried integrons. In addition, two different types of gene cassettes were detected. What’ more, MLST indicated that some MDR *K. pneumonia* strains had the same origin. To the best of our knowledge, MDR and a diversity of ARGs and MGEs existed in *K. pneumonia* isolates from captive giant pandas, and integrons were related to the emergence of MDR strains, and giant pandas may share the MDR *K. pneumonia* isolates. Therefore, it is essential that relevant precautionary measures be taken to prevent the spread of MDR bacteria, and monitor the emergence of MGEs integrons. Further studies are required to assess other factors involved in the transmission of linked resistance genes, and efforts should be made to eliminate or reduce drug resistance in captive giant pandas.

## Data Availability Statement

The datasets presented in this study can be found in online repositories. The names of the repository/repositories and accession number(s) can be found in the article/Supplementary Material.

## Ethics Statement

The animal study was reviewed and approved by the Institutional Animal Care and Use Committee (IACUC) of the Chengdu Research Base of Giant Panda Breeding (No. 2018017).

## Author Contributions

XY, JD, ZR, XS, and RH contributed to conception and design of the study. SL and JD played a guiding role in carrying out the experiment. XY, YL, DZ, CY, and LL collected samples. XY and MY performed bacterial identification and isolation and related component testing. XY, XS, HD, YD, ZX, and XF performed the statistical analysis. XY wrote the first draft of the manuscript. All authors contributed to manuscript revision, read, and approved the submitted version.

## Conflict of Interest

The authors declare that the research was conducted in the absence of any commercial or financial relationships that could be construed as a potential conflict of interest.

## Publisher’s Note

All claims expressed in this article are solely those of the authors and do not necessarily represent those of their affiliated organizations, or those of the publisher, the editors and the reviewers. Any product that may be evaluated in this article, or claim that may be made by its manufacturer, is not guaranteed or endorsed by the publisher.

## Footnotes

1
http://blast.ncbi.nlm.nih.gov

2
http://bigsdb.pasteur.fr/klebsiella/klebsiella.html
